# Multiple microarray analyses identify key genes associated with the development of Non-Small Cell Lung Cancer from Chronic Obstructive Pulmonary Disease

**DOI:** 10.7150/jca.51264

**Published:** 2021-01-01

**Authors:** Lemeng Zhang, Jianhua Chen, Hua Yang, Changqie Pan, Haitao Li, Yongzhong Luo, Tianli Cheng

**Affiliations:** Thoracic Medicine Department 1, Hunan Cancer Hospital, Changsha, Hunan Province, P.R. China, 410013.

**Keywords:** chronic obstructive pulmonary disease, non-small cell lung cancer, differentially expressed genes, module, prognosis

## Abstract

**Introduction:** Chronic obstructive pulmonary disease (COPD) is an independent risk factor of non-small cell lung cancer (NSCLC). This study aimed to analyze the key genes and potential molecular mechanisms that are involved in the development from COPD to NSCLC.

**Methods:** Expression profiles of COPD and NSCLC in GSE106899, GSE12472, and GSE12428 were downloaded from the Gene Expression Omnibus (GEO) database, followed by identification of the differentially expressed genes (DEGs) between COPD and NSCLC. Based on the identified DEGs, functional pathway enrichment and lung carcinogenesis-related networks analyses were performed and further visualized with Cytoscape software. Then, principal component analysis (PCA), cluster analysis, and support vector machines (SVM) verified the ability of the top modular genes to distinguish COPD from NSCLC. Additionally, the corrections between these key genes and clinical staging of NSCLC were studied using the UALCAN and HPA websites. Finally, a prognostic risk model was constructed based on multivariate Cox regression analysis. Kaplan-Meier survival curves of the top modular genes on the training and verification sets were generated.

**Results:** A total of 2350, 1914, and 1850 DEGs were obtained from GSE106899, GSE12472, and GSE12428 datasets, respectively. Following analysis of protein-protein interaction networks, the identified modular gene signatures containing H2AFX, MCM2, MCM3, MCM7, POLD1, and RPA1 were identified as markers for discrimination between COPD and NSCLC. The modular gene signatures were mainly enriched in the processes of DNA replication, cell cycle, mismatch repair, and others. Besides, the expression levels of these genes were significantly higher in NSCLC than in COPD, which was further verified by the immunohistochemistry. In addition, the high expression levels of H2AFX, MCM2, MCM7, and POLD1 correlate with poor prognosis of lung adenocarcinoma (LUAD). The Cox regression prognostic risk model showed the similar results and the predictive ability of this model is independent of other clinical variables.

**Conclusions:** This study revealed several key modules that closely relate to NSCLC with underlying disease COPD, which provide a deeper understanding of the potential mechanisms underlying the malignant development from COPD to NSCLC. This study provides valuable prognostic factors in high-risk lung cancer patients with COPD.

## Introduction

The morbidity and mortality of lung cancer remain on the top of malignant diseases worldwide. It has been reported that 2.1 million new lung cancer patients were associated with 1.8 million deaths in 2018, which corresponds almost to 18.4% of cancer related deaths 1. Non-small cell lung cancer (NSCLC) accounts for about 80% of lung cancers, with the majority of the cases first diagnosed at advanced stages and with 5-year survival rate less than 20% 2.

Chronic obstructive pulmonary disease (COPD) is characterized by emphysema and air-flow limitation of lung 3, 4. As an independent risk factor for developing lung cancer 5, COPD is a common comorbidity in lung cancer patients, characterized with variable prevalence in different studies from 28% to 40% 6. Both COPD and lung cancer share common pathogenesis factors, such as tobacco smoking, gene methylation, environmental factors, and chronic inflammation 7. The long-term airway inflammation of COPD patients possibly leads to the changes of the bronchial epithelium, which initiates carcinogenesis 8. Sandri et al. identified the gene expression patterns that distinguish COPD cohort in patients with or without lung cancer, suggesting that extracellular matrix (ECM) and PI3K-Akt signaling pathways are potentially involved in the malignant transformation, as these pathways were over-represented in the tumor and adjacent tissue but not in normal tissues 9. However, diverse studies have yielded diverse results and the global perspective of genomic changes from COPD to lung cancer is still unclear. Thus, this study aimed to identify the specific genetic signatures on the transcriptome level in the COPD-cohort that promote lung cancer.

Based on the high-throughput sequencing and microarray technologies, the expression changes of the genes in different samples under certain biological conditions can be simultaneously observed. Microarray profiling has shown great potential in obtaining and understanding of the molecular mechanisms by analysis of thousands of genes 10, 11. There are many studies describing the usage of integrated bioinformatics approach of multiple datasets for the identification of reliable and reproducible genetic changes involved in tumorigenesis 12, 13.

Three data sets with a large sample size, including GSE106899 14, GSE12472 15, and GSE12428 16, were analyzed in this study. Due to the complex evolutionary mechanism from COPD to lung cancer, a multi-omic approach was performed to identify gene expression patterns in COPD stroma and NSCLC. Besides, based on the module gene signatures, a prognostic risk model was constructed by using the multivariate Cox regression analysis. This study may provide deeper understanding of the potential mechanisms involved the development of NSCLC from COPD, providing meaningful factors for surveillance of NSCLC in high-risk COPD patients.

## Materials and Methods

### Retrieval of microarray datasets and the workflow of this study

**Figure 1:**
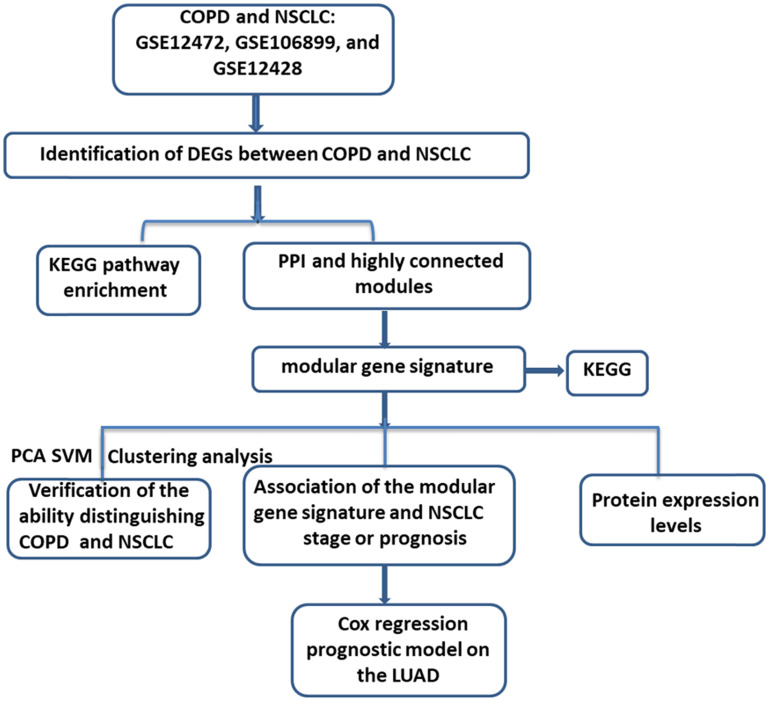
The workflow of the study. COPD, Chronic obstructive pulmonary disease; NSCLC, non-small cell lung cancer; KEGG, Kyoto Encyclopedia of Genes and Genomes; PPI, protein-protein interaction; PCA, principal component analysis; SVM, Support vector machines; LUAD, lung adenocarcinoma.

“COPD” and “Lung cancer” were sued as the key words for searching and retrieving the transcriptome profiles of COPD and lung cancer datasets from the NCBI GEO database (http://www.ncbi.nlm.nih.gov/geo/) 17. Microarray data that met the following criteria were included in the next study: 1) studies contained both COPD and Lung cancer tissues; 2) Homo sapiens origin. After further manual retrieval, three data sets were finally incorporated in our study, GSE12472, GSE106899 and GSE12428. All of the datasets contained both COPD and NSCLC samples. As shown in Figure 1, upon downloading of the expression profiles of these three datasets, the differentially expressed genes (DEGs) between COPD and NSCLC were identified. Then, based on the DEGs in the intersection by Veen analysis, the Kyoto Encyclopedia of Genes and Genomes (KEGG) enrichment analysis and protein-protein interaction (PPI) network were performed. After identification of the modules with higher gene overlap, the shared modular gene signatures were considered to play a significant role in the development from COPD to NSCLC. Subsequently, the ability of the modular gene signatures to distinguish between COPD and NSCLC samples was validated by principal component analysis (PCA), cluster analysis, and support vector machines (SVM) analysis. Finally, based on the multivariate Cox regression analysis, a prognostic risk model for NSCLC was established.

### Data preprocessing

GSE106899 contained 22 COPD samples and 22 NSCLC samples, which were sequenced by the Illumina HiSeq 2000. GSE12472 contained 10 normal samples, 18 COPD samples, and 18 NSCLC samples. Microarray data of GSE12428 included 18 COPD samples and 18 NSCLC samples. All of the microarray data of the GSE12472 and GSE12428 were detected by the Agilent-012391 Whole Human Genome Oligo Microarray G4112A (Feature Number version) platform. All the data were downloaded in August 2019.

For GSE106899, the original count matrix of the genes was first obtained, and then the raw count was normalized using the topological map-matching (TMM) algorithm in the edgeR package (Version: 3.4, http://www.bioconductor.org/packages/release/bioc/html/edgeR.html) 18, 19. Subsequently, the normalized data was converted to logCPM values for further analysis. For the GSE12472 and GSE12428, the annotation files of the platform were firstly downloaded and followed by matching the probe number and the Gene symbol one by one. The probes that do not match to the Gene symbol were removed. When different probes map to the same gene, the mean values of the different probes were considered as the final expression rate of the gene. Then, the expression matrices of GSE12472 and GSE12428 were obtained.

### Identification of the DEGs between COPD and NSCLC

For GSE106899, based on the logCPM values, the DEGs between COPD and COPD with NSCLC were identified by the edgeR package. For GSE12472 and GSE12428, the classical Bayesian method provided by the limma package was used to analyze the DEGs 20 (Version 3.10.3, http://www.bioconductor.org/packages/2.9/bioc/html/limma.html). The Benjamini & Hochberg method was used to perform multiple test calibration on the p.value and logFC values of the above genes to obtain the corrected p value (adj.P.Value). The adj.P.Val<0.05 and |logFC|>0.585 were selected as the threshold for the following analysis.

### Veen analysis of the DEGs

The DEGs in the intersections of GSE106899 *vs.* GSE12428, GSE106899 *vs.* GSE12472, and GSE12428 *vs.* GSE12472 were identified for the further analysis. The intersections of any two data sets were used as the training set and the remaining data was used as the verification set.

### Pathway enrichment analysis

The KEGG pathway enrichment analysis was performed on the three intersection DEGs using the R-package clusterProfiler (version:3.8.1, http://bioconductor.org/packages/release/bioc/html/clusterProfiler.html) 21, 22. A path with p.value < 0.05 was considered to be a result with significant enrichment.

### Construction of the Protein- Protein Interaction (PPI) Network

Based on the STRING database (Version: 10.0, http://www.string-db.org/), the interactions between gene-encoded proteins can be predicted 23. For the DEGs in the intersection of GSE106899 vs. GSE12472 and GSE106899 vs. GSE12428, the PPI score was set as 0.4 (medium confidence). Due to the large number of DEGs in the intersection of GSE12472 vs. GSE12428, the PPI score was set to 0.9 (highest confidence) in order to find a more closely interacting protein. Then, the Cytoscape software (version:3.4.0, http://chianti.ucsd.edu/cytoscape-3.4.0/) was used for visualization of the structure of PPI networks 24.

### Identification of the modular gene signatures of the PPI networks

In the PPI network, cluster analysis was applied to identify the functional modules. The MCODE plug-in of Cytoscape software with the default parameters (Degree Cutoff = 2, Node Score Cutoff = 0.2, K-core = 2, and Max.Depth = 100) was used for obtaining three key modules with high gene duplication in three PPI networks, respectively 25. The genes in the intersection of the three modules were considered to be closely related to the pathological process. Then, KEGG pathway enrichment analysis was carried out on the key genes of the module, using the R-package clusterProfiler. The pathway with *p* value < 0.05 was regarded as a pathway with significant enrichment.

### Verification of the identified modular gene signatures for discriminating COPD from NCLSC

For the further evaluation and verification of the key genes in terms of clear distinguishing between COPD and lung cancer samples, principal component analysis (PCA), cluster analysis, and support vector machines (SVM) analysis were performed. For PCA, the prcomp function (https://stat.ethz.ch/R-manual/R-devel/library/stats/html/prcomp.html) was used to reduce the dimension of the data, and the PCA map was constructed by the ggbiplot package (Version: 0.4.5, https://mirrors.tuna.tsinghua.edu.cn/CRAN/bin/windows/contrib/3.4/ggfortify_0.4.5.zip). After PCA analysis, the first two principal components were selected, and the distribution of the sample on the two-dimensional plane was mapped, according to the score of the principal component 26. The classification of the sample can be visually seen by the PCA graph. Besides, we also performed cluster analysis. For that, the distance between two samples was firstly calculated based on the expression data of key genes in the module. Then, the clust function of R language was used to analyze the samples, based on the distance between samples. Finally, the clustering results were displayed in a tree diagram. In addition, based on the first two principal components obtained by PCA analysis, the SVM classifier was constructed and the performance of the classifier was evaluated by the receiver operating characteristic (ROC) curve. Totally, PCA, cluster analysis, and SVM analyses were performed for each of the three datasets.

### Expression levels of the modular gene signatures in the GSE12472 data set

The dataset GSE12472, which contained 10 normal, 18 COPD, and 18 NSCLC samples, was used for the evaluation of expression levels of identified key genes in the normal, COPD, and NSCLC datasets. Firstly, difference analysis was performed for COPD vs. Normal and NSCLC vs. Normal based on the expression matrix of GSE12472. Then, the multiple test calibration of p.value was performed by the BH method to obtain the corrected p-value (adj.P.Value). Based on adj.P.Value <0.05 and |logFC|>0.585, the expression levels of samples were shown in the heat map.

### Survival analysis of modular gene signatures in the NSCLC

GEPIA (http://gepia.cancer-pku.cn/) is an online tool for visual analysis based on TCGA data 26. The identified modular gene signatures were used as the input, and lung squamous cell carcinoma (LUSC) and lung adenocarcinoma (LUAD) were selected to analyze the correlation between key genes and prognosis in lung cancer. The samples were divided into high and low expression groups based on the median value of Transcripts Per Million (TPM) expression of modular gene signatures for Kaplan-Meier survival analysis. At the same time, the logRank test was performed to obtain significant *P* values. The genes with the *P* value<0.05 were considered to be associated with prognosis.

### Protein expression levels of key genes in lung and lung cancer tissues

Based on the Human Protein Atlas (HPA) database (https://www.proteinatlas.org/), expression of certain proteins in different tissues and organs can be studied at the RNA and protein levels 27. In this study, the protein levels of the key genes in the lung and lung cancer tissues were investigated based on the HPA database.

### Construction of the prognostic risk model based on the Cox regression analysis

Sample clinical phenotypic information, RNA-seq data, and sample survival information for TCGA LUAD were downloaded from UCSC Xene database (https://xenabrowser.net/datapages/) 28. LUAD samples with both survival and RNA-seq information were selected for the further study. After converting the ensemble IDs into gene symbols, the expression values of modular gene signatures were selected for the further study.

Multivariate Cox regression analysis was performed on the modular gene signatures, and the prognosis risk model was constructed according to the following formula:


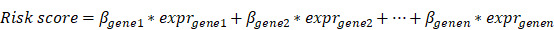


where β in the formula is a prognostic correlation coefficient of each gene of multivariate Cox regression, and “expr” is the expression value of the gene. Thus, each sample was characterized by a particular risk score. Then, optimal cut-point was determined based on the survminer of the R package (version 0.4.3). According to the risk score level in regard to the optimal cut-point, the samples were divided into high (High risk) and low risk groups (Low risk). Combined with the data on total survival time of the corresponding patients, the log-rank test was used to perform K-M survival analysis and the survival curve was generated. In addition, the expression levels of the modular gene signatures of samples were shown in the heat map. About 3/4 samples were randomly selected as the training set, and the remaining 1/4 samples were used as the verification set to construct the above model.

### Univariate and multivariate Cox regression analysis of clinical features

In order to determine whether the predictive ability of the above prognostic model can be independent of other clinical factors, the risk group, age, gender, age of tobacco smoking, and clinical stage were used as variables, and Univariate and multivariate Cox regression analyses were carried out respectively.

### Correlations between modular gene signatures and clinical factors of NSCLC, and the expression levels of modular gene signatures in other cancers

In order to explore whether key genes were associated with clinical pathological staging, their expression levels in different clinical stages of LUSC and LUAD were analyzed by UALCAN. UALCAN (http://ualcan.path.uab.edu/index.html) is a website for effective online analysis of cancer, based on The Cancer Genome Atlas (TCGA) database 29. We suggested that the above-mentioned identified key genes can be potentially related to lung cancer lesions. Besides, many reports have shown that tobacco smoking was responsible for the majority of lung cancer cases 30, 31. Therefore, the expression levels of key genes in smokers and non-smokers of LUAD and LUSC were also considered. In this part, the expression levels of key genes were shown in boxplots. In addition, based on TCGA and GEPIA, the expression levels of modular gene signatures in cancer and adjacent tissues of 31 cancers were explored.

## Results

### Identification of DEGs between COPD and NSCLC

Based on the methods above, total of 2350, 1914, and 1850 DEGs (adj.P.Val<0.05 and |logFC|>0.585) were identified between COPD and NSCLC in GSE106899, GSE12472, and GSE12428, respectively. Compared with tissues from COPD, there were 844 up-and 1506 down-regulated genes in NSCLC samples in GSE106899. For other two datasets, there were 747 up- vs. 1167 down- (GSE12472) and 785 up- vs. 1065 down- (GSE12428) regulated genes.

### Functional enrichment based on the DEGs

**Figure 2:**
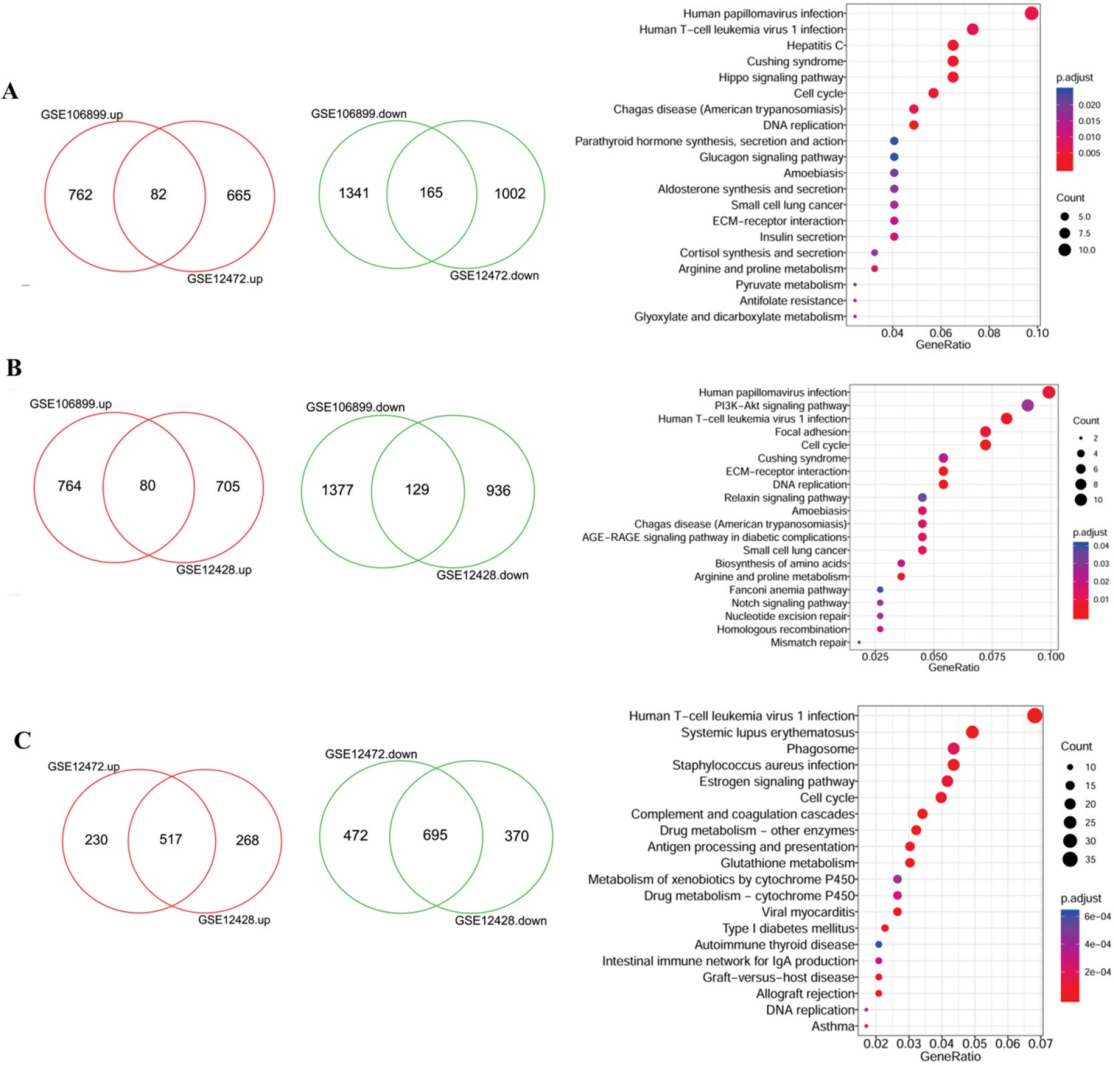
The Veen diagrams of DEGs and their enriched KEGG items. The two Veen diagrams on the left show the DEGs between COPD and COPD with lung cancer. The diagrams on the right represent the top 20 enriched KEGG pathways of DEGs. A: the DEGs of intersection of GSE106899 and GSE12472; B: the DEGs of intersection of GSE106899 and GSE12428; C: the DEGs of intersection of GSE12472 and GSE12472. Compared with COPD, the up-regulated genes in COPD lung cancer are represented in red, and the down-regulated genes are shown in green (Veen diagram). For the KEGG pathway diagram (right), GeneRation indicates the ratio of the number of genes enriched within a particular pathway to the number of input genes. Besides, the size of bubble indicates the number of enriched genes, and red gradient reflects the significance of the pathway enrichment.

Due to the large number of the DEGs, two datasets were randomly selected for intersection analysis and the identified DEGs were further subjected to functional analysis. As shown in the Figure 2A, there were 82 up- and 165 down- regulated DEGs were shared by GSE106899 and GSE12472, which were mainly enriched in 33 KEGG pathways. Besides, the DEGs in the intersection of GSE106899 and GSE12428 were mainly enriched in 20 KEGG pathways (Figure 2B). For GSE12472 and GSE12428, the shared DEGs (517 up- and 695 down-regulated genes, Figure 2C) were mainly distributed in 63 KEGG pathways, and the top 20 items are shown in the Figure 2C. Among the pathways, the top and shared pathways are Human papillomavirus infection, Human T-cell leukemia virus 1 infection, Cell cycle, DNA replication, and others. The top 20 KEGG pathways are shown in detail in Supplementary Table 1.

### PPI network and modular gene signatures involved in the NSCLC

**Figure 3:**
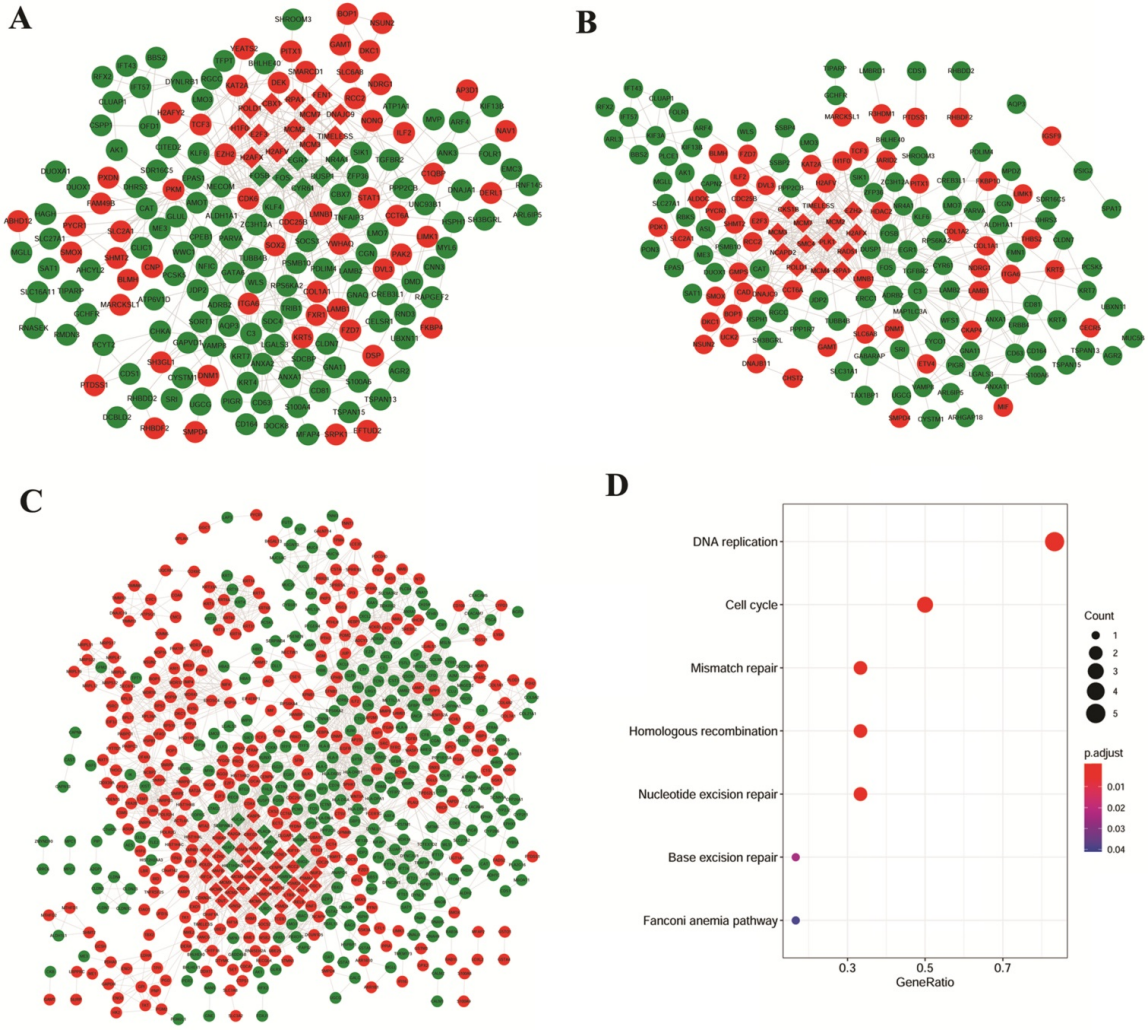
PPI networks and KEGG pathways of modular gene signatures. Red indicates up-regulated proteins and green indicates down-regulated proteins. Diamond indicates key module protein and gray line indicates interaction between proteins. A: Based on the interaction of GSE106899 and GSE12472; B: Based on the DEGs in the interaction of GSE106899 and GSE12428; C: Based on the DEGs of interaction of GSE12472 and GSE12428. D: Enriched KEGG pathways of modular gene signatures.

Based on the studies of GSE106899 and GSE12472, interaction pairs involving 196 proteins nodes were found, based on the STRING database (Figure 3A). Meanwhile, for the DEGs in the interaction maps of GSE106899 and GSE12428, total of 169 proteins were identified and 444 interaction pairs were predicted (Figure 3B). In addition, 2699 interaction pairs and 616 proteins were obtained from the interaction tests of GSE12472 and GSE12428 (Figure 3C).

Due to the large number of the protein nodes and intricate network of functions of the PPI networks, we further explored the key modules involved in the COPD with NSCLC. Based on the MCODE plugin of the Cytoscape software with the default value, several modules were obtained (the diamond nodes in Figure 3). By comparing the nodes of these modules, there were 6 shared proteins, namely H2A histone family, member X (H2AFX), mini-chromosome maintenance (MCM) proteins MCM2, MCM3, MCM7, DNA polymerase δ catalytic subunit gene (POLD1), and Replication protein A1 (RPA1). As shown in the Figure 3D, these genes were mainly enriched in the DNA replication, cell cycle, mismatch repair, homologous recombination, and nucleotide excision repair.

### Verification of the identified modular gene signatures for discriminating COPD from NCLSC

**Figure 4:**
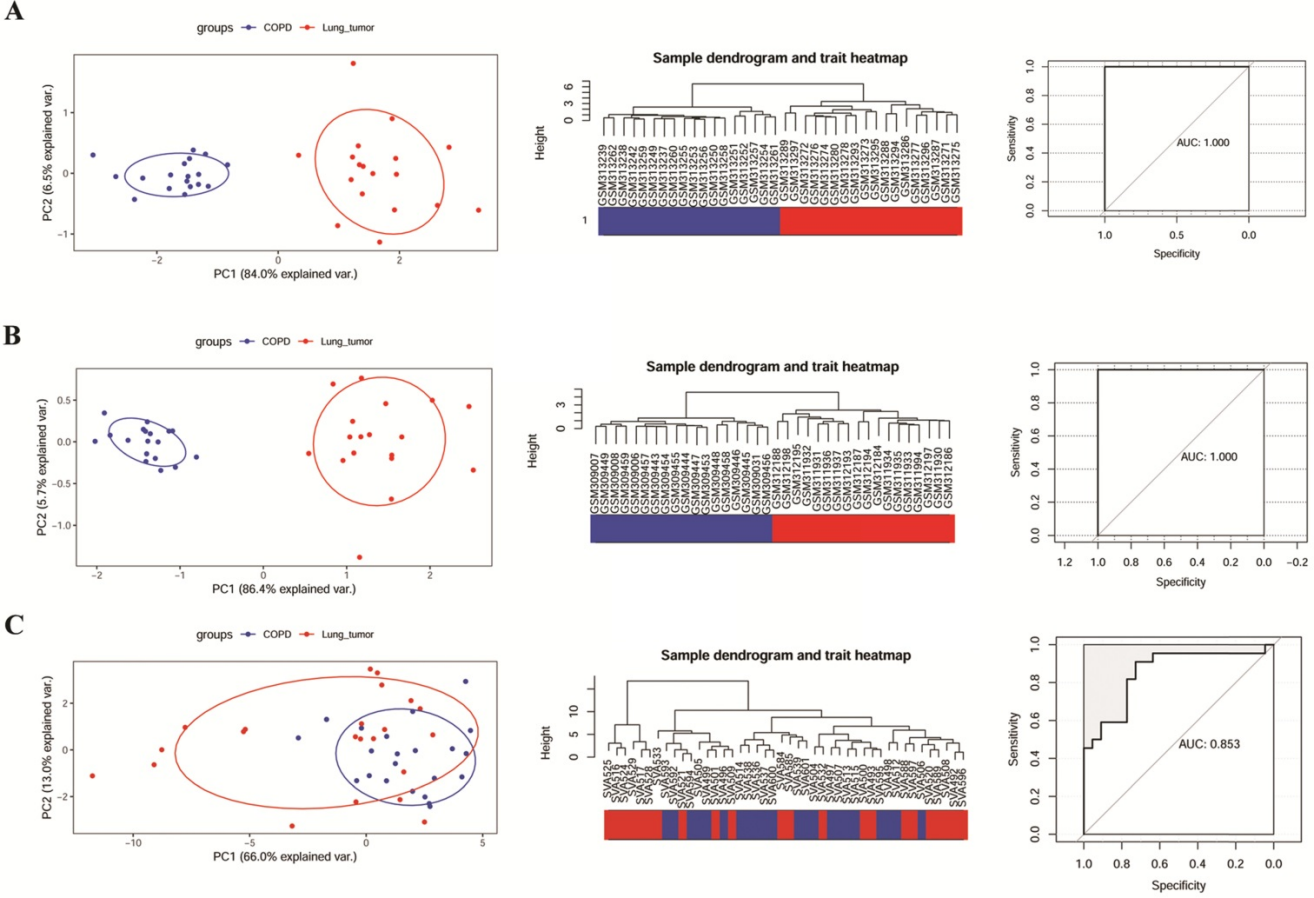
The ability of modular gene signatures to distinguish COPD from NSCLC samples. A: GSE12472; B: GSE12428; C: GSE106899. The left diagrams are PCA ordination diagrams. The middle diagrams are clustering trees. The right diagrams represent ROC curves.

The ability of 6 key genes to distinguish COPD from NCLSC samples was tested in GSE106899, GSE12472, and GSE12428. As shown in the Figure 4A and B, COPD and COPD with LUSC samples in GSE12472 and GSE12428 could be clearly distinguished via these six key genes, applying the area under the curve (AUC) of 1.000 in the ROC curves. However, these six genes did not completely distinguish between COPD and COPD with LUAD samples in GSE106899 (Figure 4C), with the AUC of 0.853 in the ROC curve.

### The expression levels of modular gene signatures in GSE12472

**Figure 5:**
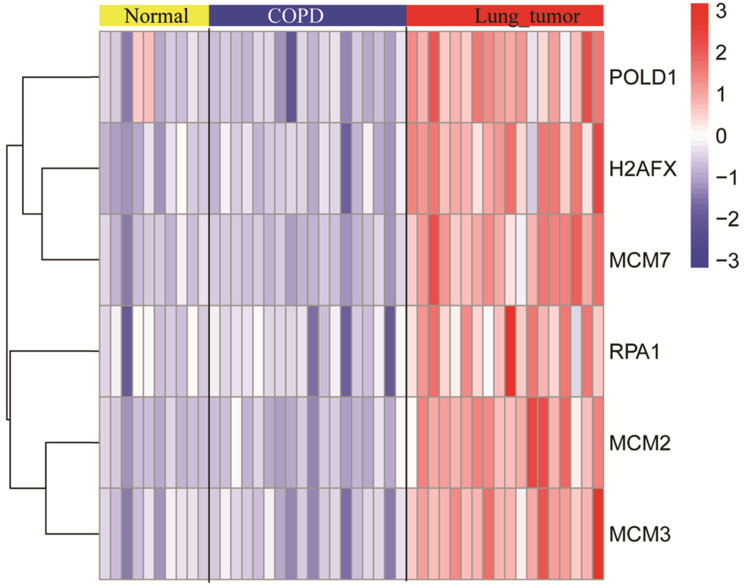
The expression levels of modular gene signatures in the normal, COPD, and NSCLC samples of GSE12472.

The expression levels of six key genes of the normal, COPD, and NSCLC samples were measured. As shown in the Figure 5, there was no significant difference in expression levels of key genes between normal and COPD samples. However, there was a significant difference between COPD and COPD with NSCLC. This result was consistent with the observations, which are shown in Figure 4.

### The modular gene signatures are closely related to prognosis of NSCLC

**Figure 6:**
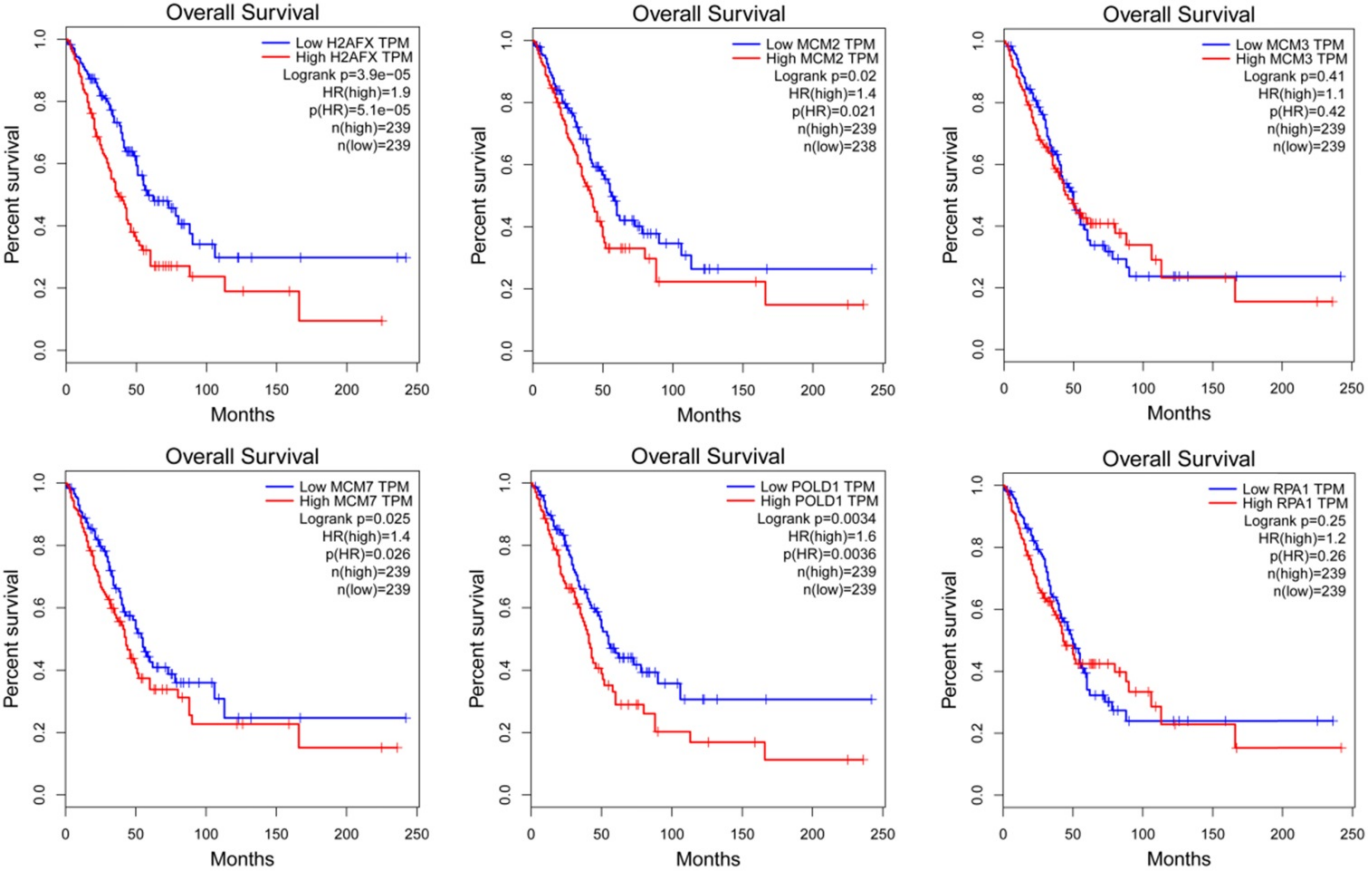
The Kaplan-Meier survival curves of modular gene signatures in LUAD sample.

The expression of modular gene signatures was significantly related to the prognosis of LUAD (*P*<0.05, Figure 6). In general, we observed that the gradually higher expressions of H2AFX, MCM2, MCM7 and POLD1 were associated with the gradually worse prognosis of LUAD. However, the expressions of six key genes were not associated with the prognosis of LUSC (Supplementary Figure 1).

### Protein expression and distribution of H2AFX, MCM2, MCM3, MCM7, RPA1, and POLD1 in lung cancer

**Figure 7:**
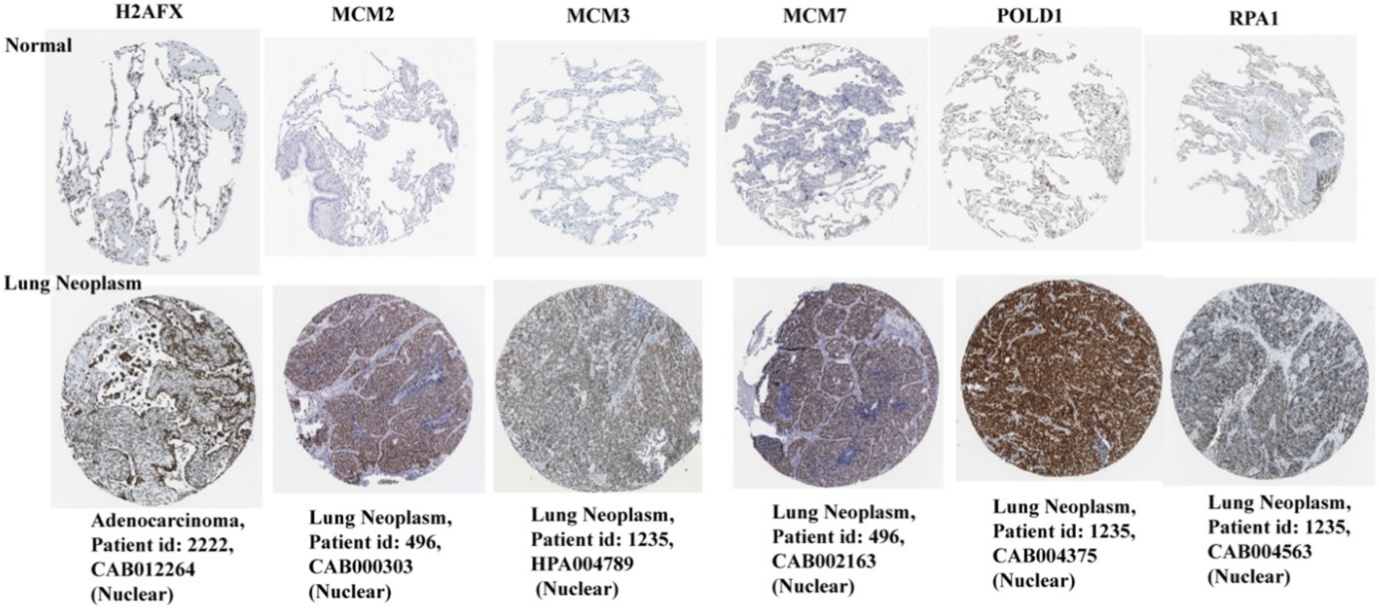
Protein immunohistochemical levels of key genes in normal lung and lung cancer tissues.

Based on the HPA database, the immunohistochemical levels of each key gene in normal lung tissue and lung cancer tissue are shown in Figure 7. It can be clearly seen that the protein levels of the six key genes were significantly elevated in lung cancer tissues compared to normal tissues.

### Construction of the prognostic risk model based on the Cox regression analysis

Based on the above results, the expression levels of H2AFX, MCM2, MCM7, and POLD1 correlate significantly with the prognosis of LUAD rather than LUSC. Thus, a prognosis model containing four genes: H2AFX, MCM2, MCM7 and POLD1, was constructed for LUAD samples. Total of 514 LUAD samples were randomly divided into training and verification sets, including 365 and 129 samples, respectively. Multivariate cox analysis was performed on the four genes in the training and validation sets, and the regression coefficients were obtained (Supplementary Table 2).

**Figure 8:**
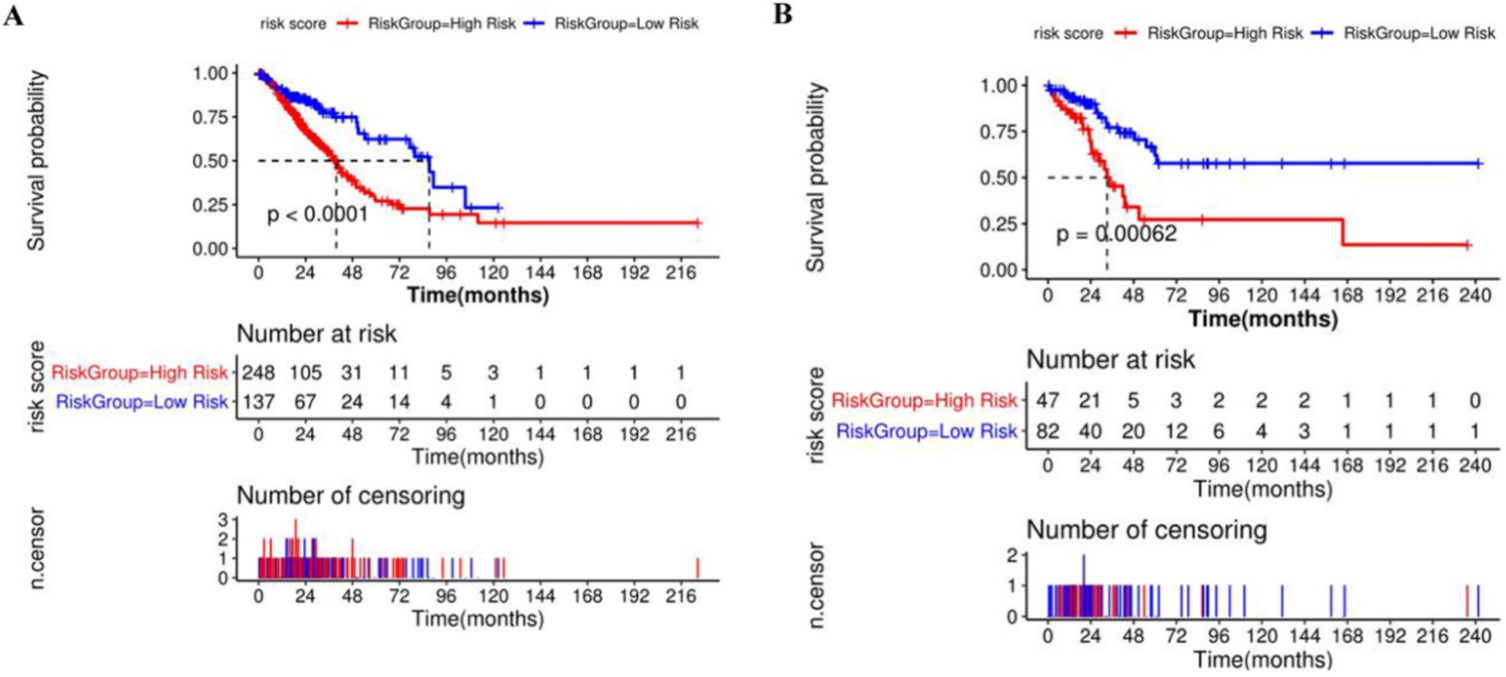
Kaplan-Meier survival curve of high and low risk groups based on Cox regression risk prognosis model.

**Figure 9:**
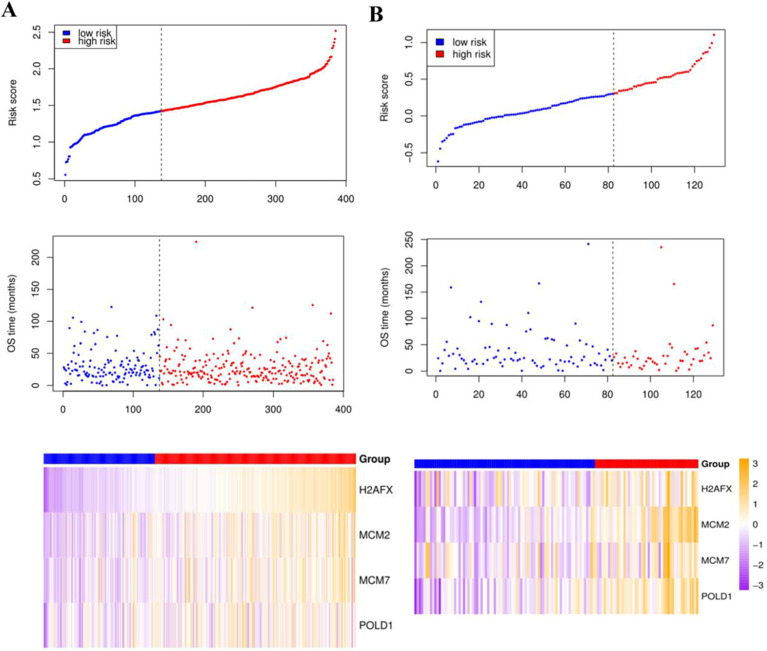
The risk score sorting scatter plot (top), scatter plot of survival time distribution (middle), and gene expression heat maps (bottom) of training (left) and verification (right) sets.

Compared with the mean and median of gene expression, optimal cutoff parameter better enables the classification between high and low risk groups 32. The optimal cutoff of the training set is 1.42, and the optimal cutoff of the verification set is 0.31 (Supplementary Figure 2). According to the optimal cutoff grouping, the K-M survival curves of the training and verification sets were obtained. Figure 8 shows that the high-risk group has a poor prognosis, both in the training and verification sets. Besides, the risk score sorting scatter plot, scatter plot of survival time distribution, and gene expression heat maps were constructed to observe the relationship between the expression levels of the four genes and the risk score. As shown in the Figure 9, the higher the gene expression, the higher the risk score and the worse is the prognosis, which is consistent with our previous results.

### Univariate and multivariate Cox regression analysis of clinical variables and risk group

**Figure 10:**
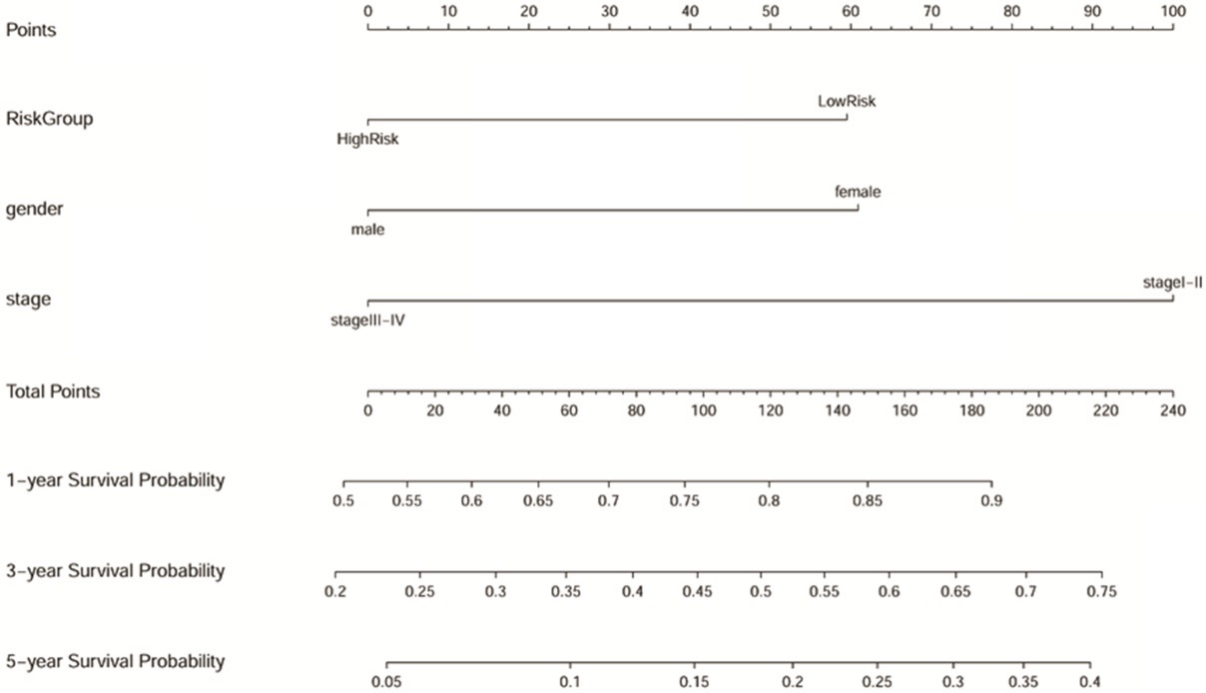
Nomogram predicting 1, 3, and 5-year overall survival for LUAD patients. The point's scale of variables was added up. The total points projected on the bottom scales indicate the probability of 1, 3, and 5-year survival.

Samples with no missing values for clinical phenotype were selected, and 193 LUAD samples were included in Cox analysis. As shown in the Table 1, gender, TNM stage, and risk group are closely related to the prognosis of LUAD both in the univariate and multivariate Cox regression analysis (p<0.05). The results suggested that the predictive power of the prognostic model can be independent of any available clinical variables (including age, gender, smoking period, and clinical stage). Besides, the Forest map (Supplementary Figure 3) and alignment Diagram (Figure 10) were also created. The nomogram made the cox regression results more straightforward. Based on the Figure 10, the points corresponding to different factors were combined for the obtaining of the corresponding survival rate.

### The modular gene signatures are closely related to clinical stages

**Figure 11:**
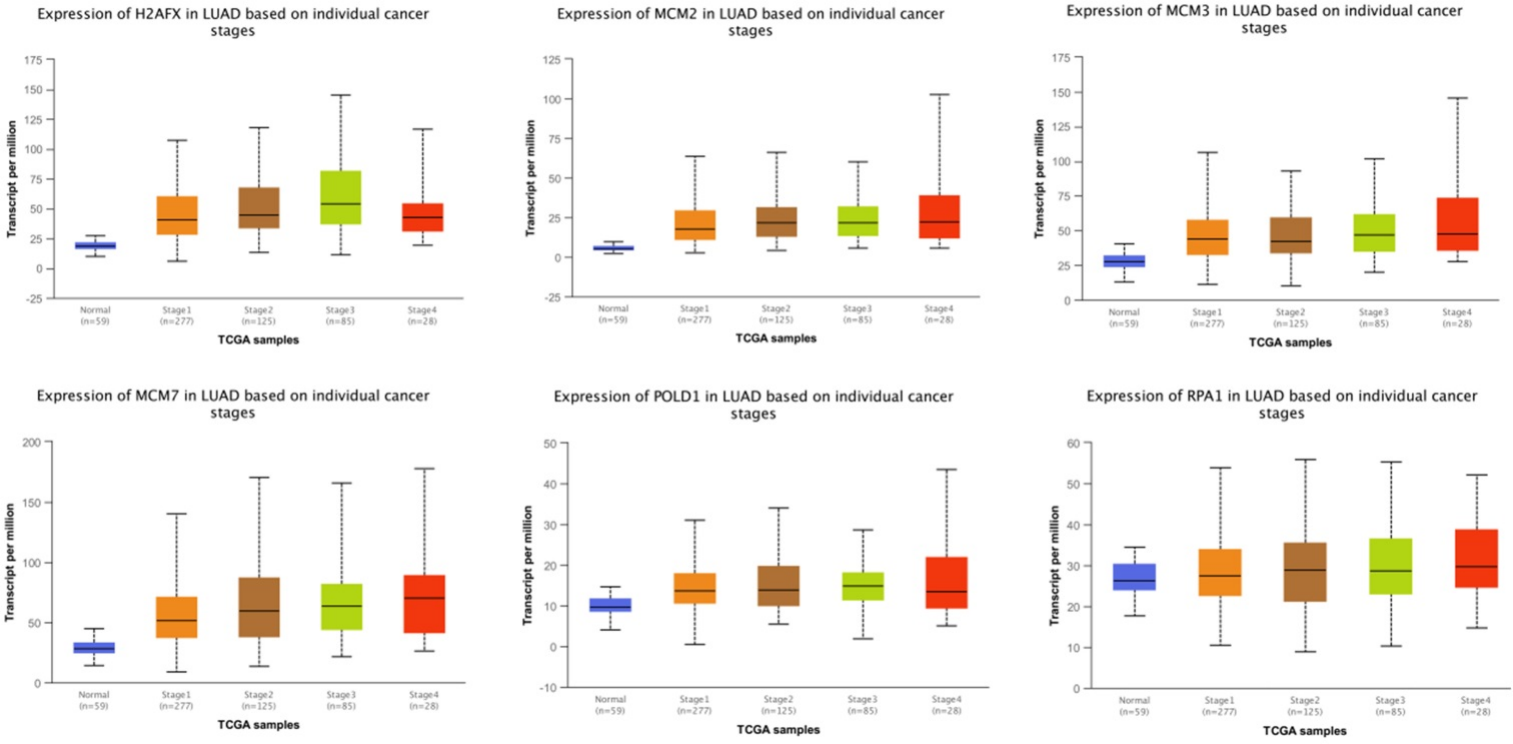
The expression levels of 6 genes at different clinical stages of LUAD.

The expressions of modular gene signatures showed an upward trend with clinical stage progression in LUAD (Figure 11), and each stage was significantly different from the normal state (Supplementary Table 3). Meanwhile, levels of modular gene signatures were upregulated in LUSC grade group, compared with the normal group (Supplementary Figure 4). In addition, H2AFX, MCM2, MCM7, and POLD1 were highly up-regulated in 31 cancer tissues, based on the analysis of TCGA data (Supplementary Figure 5). Interestingly, the expression levels of modular gene signatures were significantly different between the smoking and non-smoking groups in LUAD patients rather than in LUSC patients (Supplementary Figure 6 and Supplementary Table 3).

## Discussion

This study aimed to identify the specific genetic signatures on the transcriptome level in the COPD cohort that promote NSCLC. Compared with similar published studies on COPD, this study systematically integrated three independent data sets which contained COPD and NSCLC samples, and had a large sample size. The results we obtained were robust and strong. Based on the shared DEGs between COPD and NSCLC samples, several highly connected modules were identified. Among of the overlapping genes, a panel of module gene signatures of MCM2, MCM3, MCM7, H2AFX, POLD1, and RPA1, which could robustly distinguish COPD from NSCLC, were considered to be highly associated with the development and prognosis of NSCLC. In addition, the predictive power of the Cox regression prognostic model containing MCM2, MCM7, H2AFX, and POLD1, can be independent of other clinical variables in LUAD.

According to KEGG pathways enrichment analysis, it is possible to systematically dissect a large number of genes that refine key related biological pathways. In our study, DEGs were found to be enriched in many infection-related pathways, such as in Human papillomavirus infection, Human T-cell leukemia virus 1 infection, and others. Human papillomaviruses refer to DNA tumor viruses that infect keratinocytes in the epithelia, which have been reported to be a high risk factor for lung cancer development 33. The oncogenicity of human papillomaviruses was mainly focused on P53, which may be inactivated by virus infection 34. Human T-cell leukemia virus 1 infection can induce T cell leukemia, megalocytic leukemia, etc. 35. Shan et al. stated that the key genes involved in human T-cell leukemia virus 1 infection pathway may play an important role in the hepatocellular carcinoma development 36. However, there were few reports about the correlation of the human T-cell leukemia virus 1 infection and lung cancer.

In our study, H2AFX, MCM2, MCM3, MCM7, POLD1, and RPA1 were associated with the development of NSCLC from COPD. The pathways, enriched by the module gene signatures, were reported to be involved in the processes of DNA replication, cell cycle and mismatch repair, which have significant role in lung carcinogenesis 37, 38. For example, the mismatch repair pathway is involved in the correcting the DNA base mismatches during DNA replication and recombination 39. Base excision repair pathway plays important role in the DNA repair process 40. Mismatch repair pathway and base excision repair pathway together with cell cycle and DNA replication pathways are determinants of the cell fate 41, 42. The Fanconi Anemia pathway is a complex mechanism containing homologous recombination, nucleotide excision repair, and mutagenic translesion synthesis 43. Saviozzi et al. reported on the upregulation of genes related to homologous recombination and DNA replication pathways in the samples of NSCLC patients, which is consistent with the results of our study 44.

A panel of module gene signatures, H2AFX, MCM2, MCM7, and POLD1, were highly related to NSCLC; and the high expression levels of MCM2, MCM7, H2AFX, and POLD1 were also correlated with poor prognosis, suggesting their potential role in tumorigenesis. MCMs are protein family with fundamental functions in the replication of eukaryotic cells and are essential for initiating the DNA replication 45. MCMs were suggested to serve as good markers of the degree of proliferation activity 46. Many studies have shown that MCMs are predictors of patient survival and biomarkers of various cancers, such as lung cancer 47, 48, bladder cancer 49, 50, prostate cancer 51, salivary gland tumors 52. Ramnath et al. showed that immunostaining of tumor cells for MCM2 is an independent prognostic factor for survival of NSCLC patients 53. Liu et al. suggested that expression of MCM7 in tumor tissues may be a potential marker of poor prognosis in patients with NSCLC, whereas overexpression of MCM7 is more common in poorly differentiated tumor tissues. 54. In our study, the expression levels of MCM2, MCM3, and MCM7 were higher in NSCLC than in COPD samples. Besides, the levels of MCM2 and MCM7 were significantly associated with the prognosis of LUAD. The results of our study are consistent with the previous reports.

Exome-sequencing results based on the TCGA analysis indicated the specific association of POLD1 mutations with hyper-mutated cancers 55. Wang et al. found that POLE/POLD1 mutations could be promising potential predictive biomarkers for positive immune-checkpoint inhibitor outcomes 56. In addition, many carcinogenic factors promote the expansion of the cancer cells through the upregulation of POLD1 57. We found that the expression of POLD1 is significantly upregulated in the NSCLC and that high POLD1 expression indicates a poor prognosis in the LUAD. As a member of the histones family, H2AFX has been reported to be involved in the DNA repair pathway 58, 59. Several studies showed the dysregulation of H2AFX in the lung cancer 60-62. However, there were few reports on the potential mechanisms underlying the effect of H2AFX in lung cancer. Further studies are required to figure out the potential role of H2AFX in NSCLC.

Our study showed that high levels of H2AFX, MCM2, MCM7, and POLD1 suggest poor prognosis of LUAD rather than LUSC. This difference may be due to the high heterogeneity between LUAD and LUSC 63. LUSC is characterized with higher background of the mutation rates than LUAD, suggesting probable difficulty in prognosis prediction of LUSC using a small number of genes 64. On the contrary, based on the expression levels of H2AFX, MCM2, MCM7, and POLD1, the Cox regression prognostic model was successfully constructed for the LUAD samples and the predictive power was independent of other clinical variables. The expression levels of gene profiles were used to predict the overall survival times of the patients. Survival prediction is usually considered as a regression problem to model patients' survival time and, thus, Cox regression models are generally used for construction of the risk prognosis model 65, 66. Our model showed a higher predictive ability both in training and testing sets in LUAD samples.

Besides, H2AFX, MCM2, MCM7, and POLD1 were highly up-regulated in 31 cancer tissues. A previous shown that MCM2 was up-regulated in cervical carcinogenesis 67. Ramsauer et al. uncovered that MCM7 expression was upregulated in upper proliferating keratinocyte layers of papillomas 68. Moreover, POLD1 was reported associated with colorectal cancer 69, 70. Interestingly, the expression levels of modular gene signatures were significantly different between the smoking and non-smoking groups in LUAD patients rather than in LUSC patients. The results were further shown that the modular gene signatures were risk genes.

However, there are still some limitations in this study. Further studies with much larger sample sizes will be needed. In addition, the data used in study are downloaded from publicly available databases, lacking of any original datum from our own study. Further validation, either *in vivo* or *in vitro* experiment will be needed. The molecular signaling pathway and function should be explored by further experiments or clinical study. Relevant experiments will be performed to verify the multiple candidate targets identified from our bioinformatics analyses.

In conclusion, the expression levels of H2AFX, MCM2, MCM7, and POLD1 were significantly different between COPD and NSCLC samples. The modular gene signatures were mainly enriched in the pathways of DNA replication, cell cycle, and mismatch repair. The high expression levels of H2AFX, MCM2, MCM7, and POLD1 were correlated with poor prognosis of LUAD.

## Supplementary Material

Supplementary figures and tables.

## Figures and Tables

**Table 1:** Univariate and multivariate cox regression analysis of clinical phenotypes on LUAD samples

Variables	Univariate analysis			Multivariate analysis		
	HR	95 % CI	*P* value	HR	95 % CI	*P* value
**Gender**						
Male/Female	2.068	1.264-3.382	0.003807702	1.852	1.124-3.054	0.01565781
**Age**						
>60/≤60	1.273	0.752-2.158	0.368987051	1.293	0.754-2.216	0.35075578
**TNM stage**						
Stage III-IV/Stage I-II	3.019	1.760-5.179	0.00006	2.414	1.374-4.241	0.00216557
Smoke years						
>15/≤15	1.194	0.569-2.503	0.639280745	0.990	0.464-2.112	0.98036512
**Risk group**						
High/Low	2.412	1.320-4.407	0.003807702	2.217	1.198-4.102	0.01124920

## Abbreviations

COPD: Chronic obstructive pulmonary disease
NSCLC: non-small cell lung cancer
GEO: Gene Expression Omnibus
DEG: differentially expressed genes
PCA: principal component analysis
SVM: Support vector machines
PPI: protein-protein interaction
KEGG: Kyoto Encyclopedia of Genes and Genomes
TCGA: The Cancer Genome Atlas
LUAD: lung adenocarcinoma
LUSC: lung squamous cell carcinoma
ECM: extracellular matrix
H2AFX: H2A histone family, member X
MCM: mini-chromosome maintenance proteins
POLD1: DNA polymerase δ catalytic subunit gene
