# Supplementary material for: Multiple microarray analyses identify key genes associated with the development of Non-Small Cell Lung Cancer from Chronic Obstructive Pulmonary Disease
Source: J Cancer. 2021 Jan 1;12(4):996–1010. doi: 10.7150/jca.51264 (PMC7797649; doi:10.7150/jca.51264)
Supplement: Supplementary file 1 — Supplementary figures and tables. [file jcav12p0996s1.pdf]

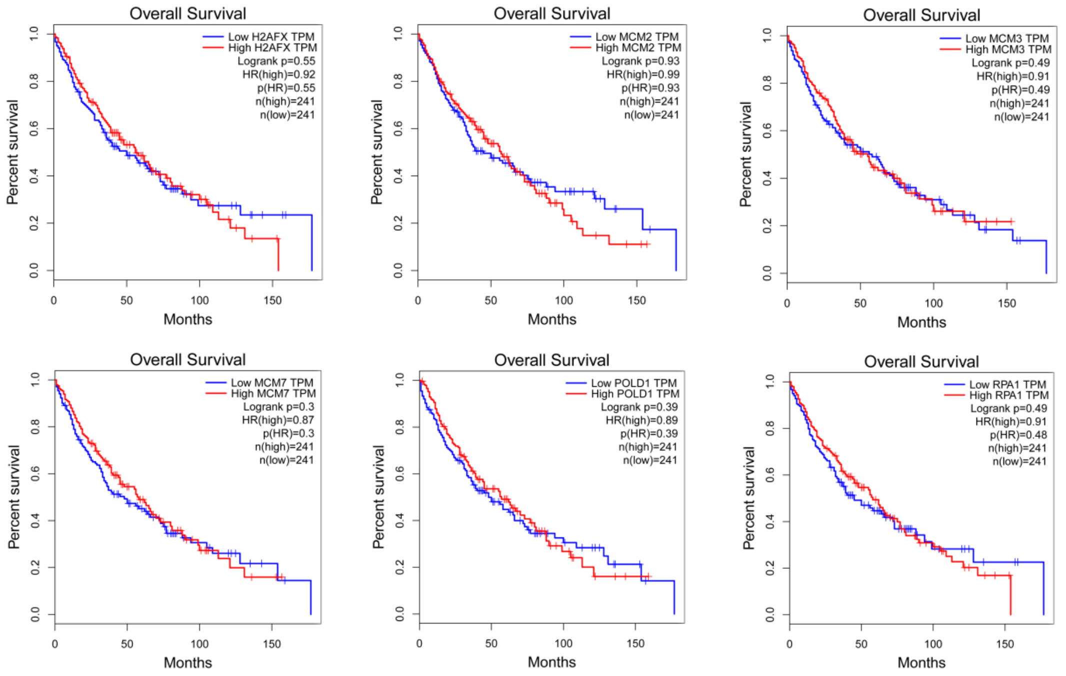

Supplementary Figure 1 The Kaplan-Meier survival curves of modular gene signatures in LUSC samples.

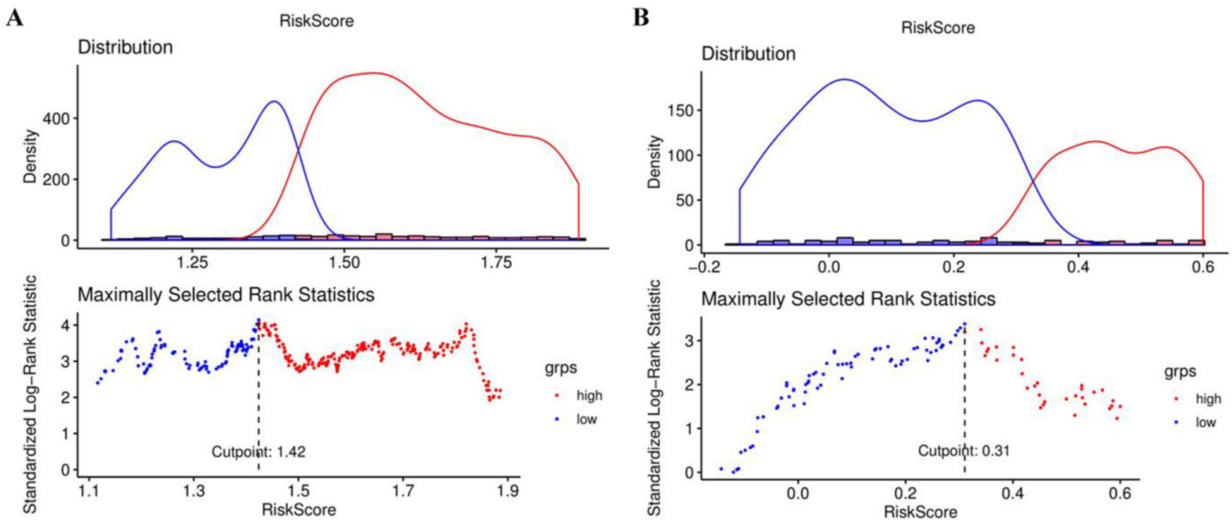

Supplementary Figure 2 The distribution of the risk score and identification of the optimal cutoff values.

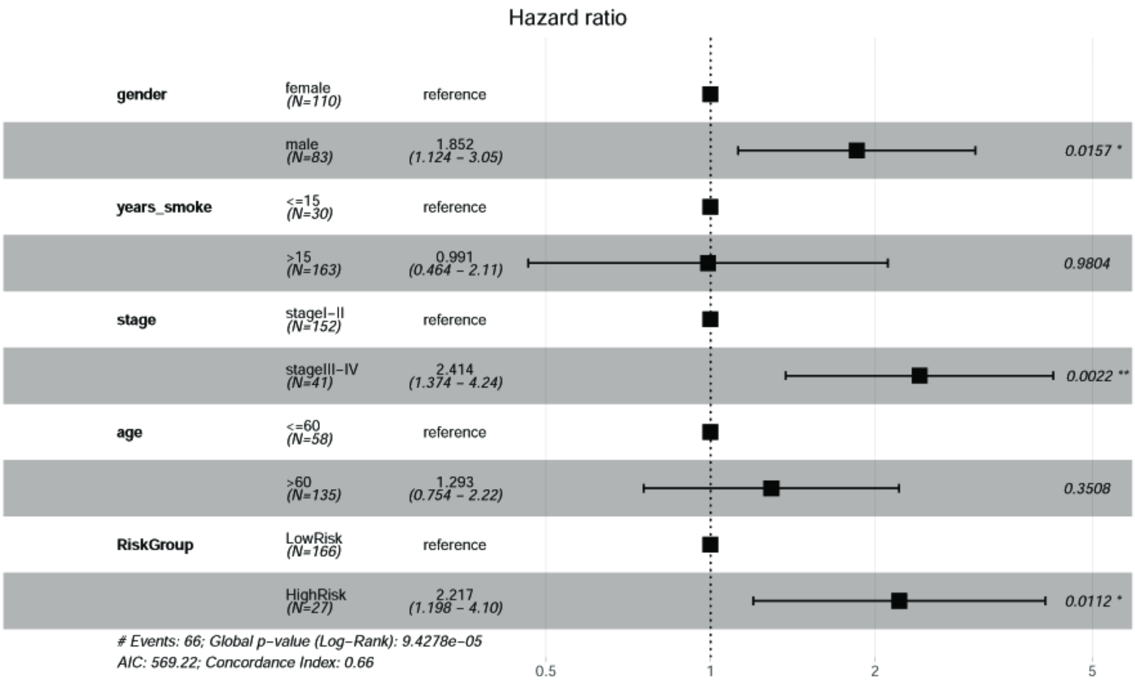

Supplementary Figure 3 Forest plot of the multivariate regression analysis based on the clinical factors of LUAD.

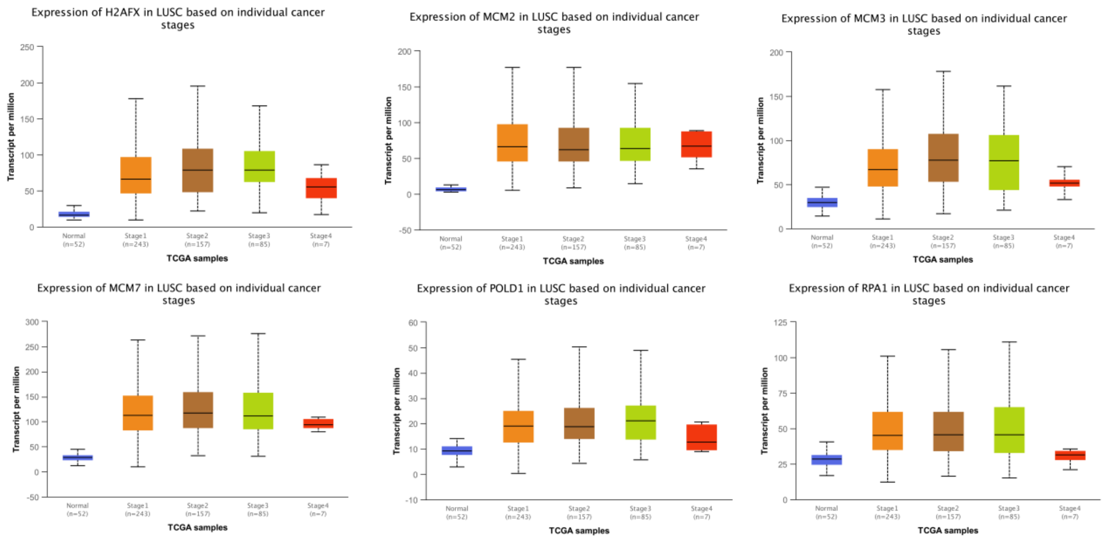

Supplementary Figure 4 The expression box plots of modular gene signatures in LUSC samples.

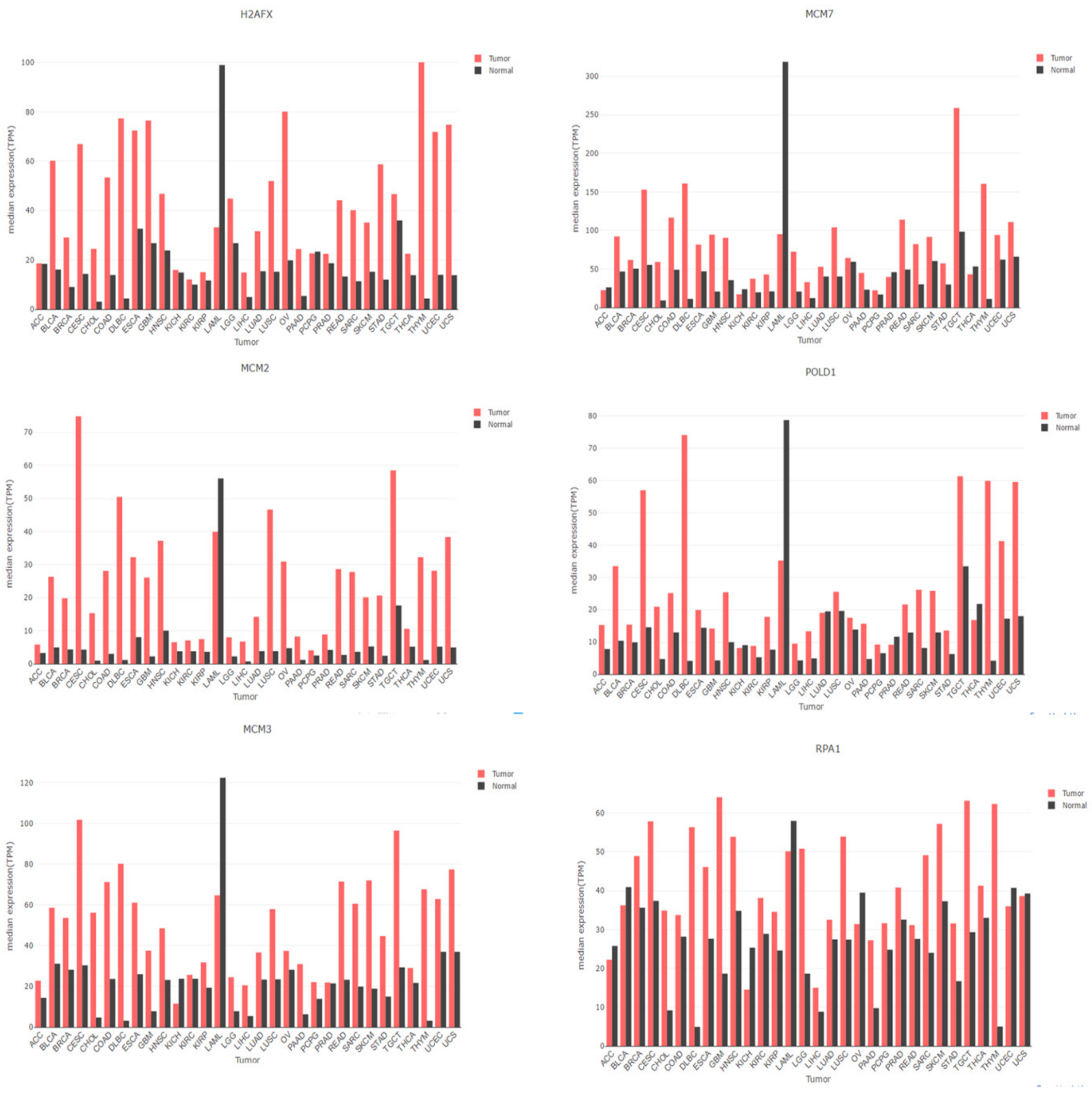

Supplementary Figure 5 Relative expression levels of modular gene signatures in other cancers (31 cancers) and corresponding adjacent tissues based on TCGA analysis.

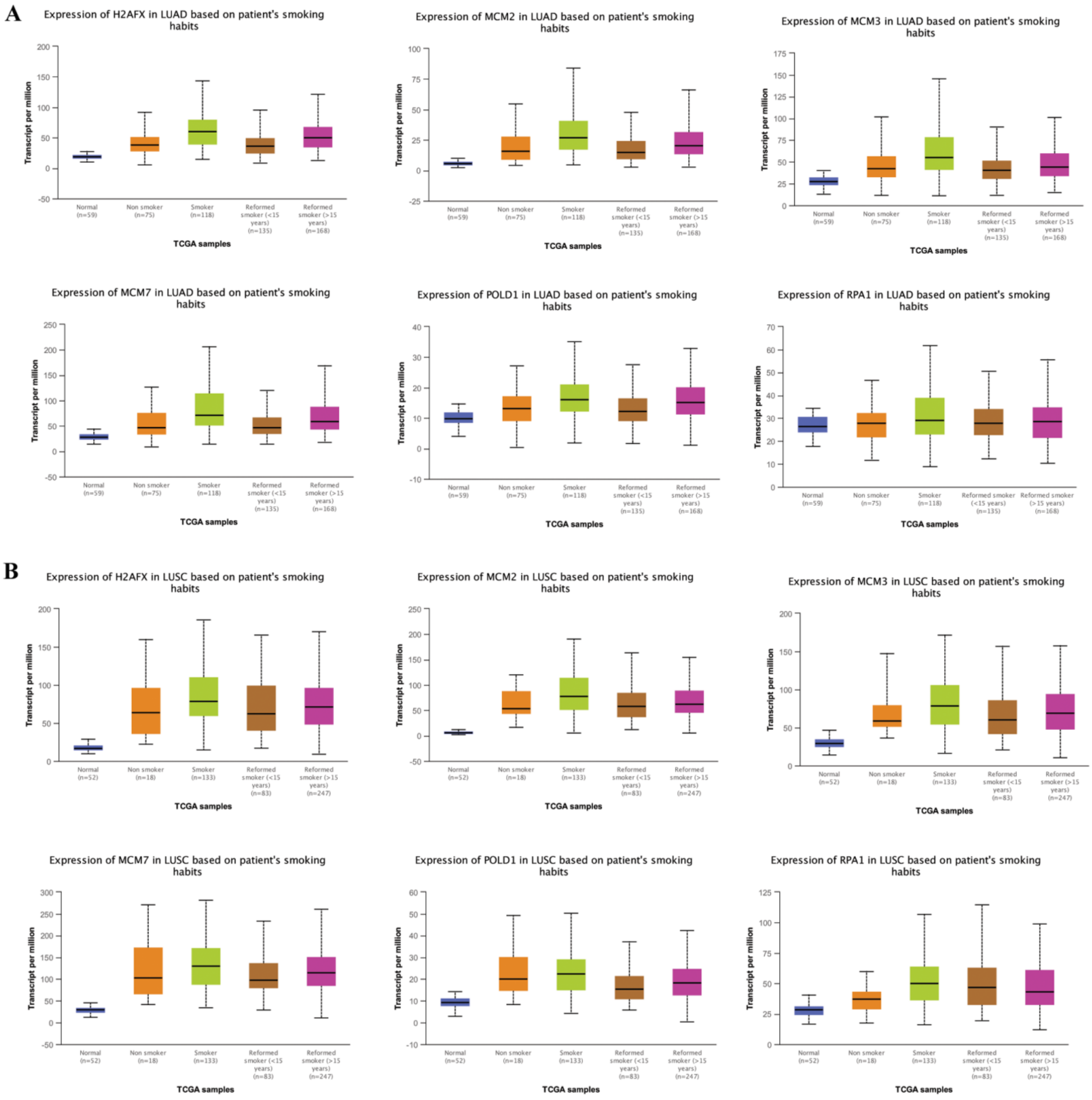

Supplementary Figure 6 Correlation between the expression levels of modular gene signatures and the smoking or non-smoking groups in LUAD and LUSC patients.

Supplementary Table 1 The top 20 enriched KEGG pathways based on the DEGs in the intersection of two datasets

Data sets	ID	Description	GeneRatio	pvalue	Count
GSE106899 vs GSE12472	hsa03030	DNA replication	6/123	1.69E-05	6
	hsa04390	Hippo signaling pathway	8/123	0.002722	8
	hsa04934	Cushing syndrome	8/123	0.002834	8
	hsa05160	Hepatitis C	8/123	0.002834	8
	hsa04110	Cell cycle	7/123	0.003153	7
	hsa05142	Chagas disease (American trypanosomiasis)	6/123	0.005067	6
	hsa05165	Human papillomavirus infection	12/123	0.005226	12
	hsa05166	Human T-cell leukemia virus 1 infection	9/123	0.007084	9
	hsa00330	Arginine and proline metabolism	4/123	0.007458	4
	hsa04911	Insulin secretion	5/123	0.010836	5
	hsa00630	Glyoxylate and dicarboxylate metabolism	3/123	0.011052	3
	hsa04512	ECM-receptor interaction	5/123	0.011894	5
	hsa01523	Antifolate resistance	3/123	0.012099	3
	hsa05222	Small cell lung cancer	5/123	0.014843	5
	hsa04925	Aldosterone synthesis and secretion	5/123	0.018247	5
	hsa04927	Cortisol synthesis and secretion	4/123	0.018353	4

	hsa05146	Amoebiasis	5/123	0.021315	5
	hsa00620	Pyruvate metabolism	3/123	0.022487	3
	hsa04922	Glucagon signaling pathway	5/123	0.024702	5
	hsa04928	Parathyroid hormone synthesis, secretion and action	5/123	0.024702	5
GSE106899 vs GSE12428	hsa03030	DNA replication	6/111	9.36E-06	6
	hsa04110	Cell cycle	8/111	0.00034	8
	hsa04512	ECM-receptor interaction	6/111	0.001441	6
	hsa05166	Human T-cell leukemia virus 1 infection	9/111	0.003617	9
	hsa00330	Arginine and proline metabolism	4/111	0.005201	4
	hsa05165	Human papillomavirus infection	11/111	0.006554	11
	hsa04510	Focal adhesion	8/111	0.006848	8
	hsa05222	Small cell lung cancer	5/111	0.009825	5
	hsa04933	AGE-RAGE signaling pathway in diabetic complications	5/111	0.013178	5
	hsa05142	Chagas disease (American trypanosomiasis)	5/111	0.014262	5
	hsa05146	Amoebiasis	5/111	0.014262	5
	hsa03440	Homologous recombination	3/111	0.01962	3
	hsa01230	Biosynthesis of amino acids	4/111	0.021022	4
	hsa04934	Cushing syndrome	6/111	0.021844	6
	hsa04151	PI3K-Akt signaling pathway	10/111	0.026981	10

	hsa03420	Nucleotide excision repair	3/111	0.028096	3
	hsa04330	Notch signaling pathway	3/111	0.02967	3
	hsa04926	Relaxin signaling pathway	5/111	0.03508	5
	hsa03460	Fanconi anemia pathway	3/111	0.040068	3
	hsa03430	Mismatch repair	2/111	0.040968	2
GSE12472 vs GSE12428	hsa05150	Staphylococcus aureus infection	23/528	5.18E-08	23
	hsa05166	Human T-cell leukemia virus 1 infection	36/528	3.65E-07	36
	hsa00480	Glutathione metabolism	16/528	4.39E-07	16
	hsa05322	Systemic lupus erythematosus	26/528	5.50E-07	26
	hsa04610	Complement and coagulation cascades	18/528	3.23E-06	18
	hsa00983	Drug metabolism - other enzymes	17/528	1.37E-05	17
	hsa04940	Type I diabetes mellitus	12/528	1.61E-05	12
	hsa05330	Allograft rejection	11/528	2.49E-05	11
	hsa05416	Viral myocarditis	14/528	3.01E-05	14
	hsa04612	Antigen processing and presentation	16/528	3.87E-05	16
	hsa05332	Graft-versus-host disease	11/528	5.44E-05	11
	hsa04110	Cell cycle	21/528	6.55E-05	21
	hsa04915	Estrogen signaling pathway	22/528	0.000112	22
	hsa05310	Asthma	9/528	0.000134	9

	hsa04145	Phagosome	23/528	0.000178	23
	hsa00982	Drug metabolism - cytochrome P450	14/528	0.000246	14
	hsa04672	Intestinal immune network for IgA production	11/528	0.000307	11
	hsa00980	Metabolism of xenobiotics by cytochrome P450	14/528	0.000442	14
	hsa03030	DNA replication	9/528	0.000463	9
	hsa05320	Autoimmune thyroid disease	11/528	0.00063	11

Supplementary Table 2 the coefficients of Cox regression analysis based on the training and validation sets

**Training set**		Validation set	
**H2AFX**	0.422	H2AFX	-0.030
**MCM2**	0.064	MCM2	0.368
**MCM7**	-0.038	MCM7	-0.360
**POLD1**	-0.174	POLD1	0.274

Supplementary Table 3 The correlation of key genes and different clinical stages of lung cancer as well as smoking

clinical stages of LUAD		Comparison	Statistical significance
	H2AFX	Normal-vs-Stage1	<1E-12
		Normal-vs-Stage2	1.62459E-12
		Normal-vs-Stage3	1.62448E-12
		Normal-vs-Stage4	3.8916E-05
	MCM2	Normal-vs-Stage1	<1E-12
		Normal-vs-Stage2	<1E-12
		Normal-vs-Stage3	1.6257E-12
		Normal-vs-Stage4	4.4625E-05
	MCM3	Normal-vs-Stage1	1.62437E-12
		Normal-vs-Stage2	2.4758E-14
		Normal-vs-Stage3	5.32907E-15
		Normal-vs-Stage4	0.00031598
	MCM7	Normal-vs-Stage1	1.62437E-12
		Normal-vs-Stage2	2.44249E-15
		Normal-vs-Stage3	1.3902E-08
		Normal-vs-Stage4	0.00010023

	POLD1	Normal-vs-Stage1	1.62437E-12
		Normal-vs-Stage2	1.68365E-12
		Normal-vs-Stage3	2.8151E-11
		Normal-vs-Stage4	0.0024187
	RPA1	Normal-vs-Stage1	7.9005E-05
		Normal-vs-Stage2	0.0054362
		Normal-vs-Stage3	0.0072421
		Normal-vs-Stage4	0.0195787
clinical stages of LUSC	H2AFX	Normal-vs-Stage1	<1E-12
		Normal-vs-Stage2	1.62437E-12
		Normal-vs-Stage3	<1E-12
		Normal-vs-Stage4	0.007936
	MCM2	Normal-vs-Stage1	<1E-12
		Normal-vs-Stage2	<1E-12
		Normal-vs-Stage3	1.62448E-12
		Normal-vs-Stage4	0.00030456
	MCM3	Normal-vs-Stage1	<1E-12
		Normal-vs-Stage2	<1E-12
		Normal-vs-Stage3	1.62759E-12

		Normal-vs-Stage4	0.0113638
	MCM7	Normal-vs-Stage1	<1E-12
		Normal-vs-Stage2	<1E-12
		Normal-vs-Stage3	<1E-12
		Normal-vs-Stage4	0.00108228
	POLD1	Normal-vs-Stage1	<1E-12
		Normal-vs-Stage2	<1E-12
		Normal-vs-Stage3	<1E-12
		Normal-vs-Stage4	0.051703
	RPA1	Normal-vs-Stage1	<1E-12
		Normal-vs-Stage2	1.62448E-12
		Normal-vs-Stage3	1.85607E-12
		Normal-vs-Stage4	0.045076
Smoking of LUAD	H2AFX	Nonsmoker-vs-Smoker	0.000140392
		Reformed smoker1-vs-Reformed smoker2	3.5097E-06
	MCM2	Nonsmoker-vs-Smoker	0.00055647
		Reformed smoker1-vs-Reformed smoker2	0.00034339
	MCM3	Nonsmoker-vs-Smoker	0.024084
		Reformed smoker1-vs-Reformed smoker2	0.22294

	MCM7	Nonsmoker-vs-Smoker	0.00023862
		Reformed smoker1-vs-Reformed smoker2	0.00120064
	POLD1	Nonsmoker-vs-Smoker	0.0013986
		Reformed smoker1-vs-Reformed smoker2	0.00186203
	RPA1	Nonsmoker-vs-Smoker	0.014041
		Reformed smoker1-vs-Reformed smoker2	0.40334
Smoking of LUSC	H2AFX	Nonsmoker-vs-Smoker	0.051965
		Reformed smoker1-vs-Reformed smoker2	0.059
	MCM2	Nonsmoker-vs-Smoker	0.54136
		Reformed smoker1-vs-Reformed smoker2	0.5216
	MCM3	Nonsmoker-vs-Smoker	0.49516
		Reformed smoker1-vs-Reformed smoker2	0.95942
	MCM7	Nonsmoker-vs-Smoker	0.36546
		Reformed smoker1-vs-Reformed smoker2	0.30354
	POLD1	Nonsmoker-vs-Smoker	0.68736
		Reformed smoker1-vs-Reformed smoker2	0.42388
	RPA1	Nonsmoker-vs-Smoker	0.7501
		Reformed smoker1-vs-Reformed smoker2	0.091836

Reformed smoker1 (who are current reformed smokers for <= 15 years), and

reformed smoker2 (who are current reformed smokers for >15 years).
